# Intestinal Microbiota Regulate Certain Meat Quality Parameters in Chicken

**DOI:** 10.3389/fnut.2022.747705

**Published:** 2022-04-25

**Authors:** Jiaqi Lei, Yuanyang Dong, Qihang Hou, Yang He, Yujiao Lai, Chaoyong Liao, Yoichiro Kawamura, Junyou Li, Bingkun Zhang

**Affiliations:** ^1^State Key Laboratory of Animal Nutrition, College of Animal Science and Technology, China Agricultural University, Beijing, China; ^2^College of Animal Science, Shanxi Agricultural University, Taigu, China; ^3^Kawamura & Co., Ltd., Tokyo, Japan; ^4^Graduate School of Agricultural and Life Sciences, The University of Tokyo, Ibaraki, Japan

**Keywords:** drip losing rate, intestinal microbiota, FMT, native breed, MYOD1

## Abstract

Growing evidence of intestinal microbiota-muscle axis provides a possibility to improve meat quality of broilers through regulating intestinal microbiota. Water-holding capacity is a crucial factor to evaluate the meat quality. High quality of water-holding capacity is usually described as a low drip-losing rate. This study aimed to explore the relationship between intestinal microbiota and water-holding capacity of muscle in broilers. According to our results, two native breeds of broilers (the Arbor Acres broilers and the Beijing-You broilers) exhibited remarkable differences in microbiota composition. However, the regular of gut bacteria compositions gradually became similar when the two breeds of broiler were raised in a same feeding environment. Therefore, this similar regular of intestinal microbiota induced similar water-holding capacity of the muscle from the two breeds. In subsequent fecal microbiota transplantation (FMT) experiments, the intestinal microbiota community of the Arbor Acres broilers was remodeling by oral gavage of bacterial suspension that was derived from the Beijing-You broilers. Then, not only body weight and abdominal fat rate were increased, but also drip loss of muscle was decreased in the Arbor Acres broilers. Additionally, muscle fiber diameter of biceps femoris muscle and expression of MyoD1 were notably enlarged. Muscle fiber diameter and related genes were deemed as important elements for water-holding capacity of muscle. Simultaneously, we screened typical intestinal bacteria in both the two native breeds of broilers by 16S rDNA sequencing. *Lachnoclostridium* was the only bacteria genus associated with drip-losing rate, meat fiber diameter, body weight, and abdominal fat rate.

**Importance:**

Higher body weight and superior meat quality in livestock imply an adequate source of protein and substantial commercial value. Regulating the intestinal microbiota of broilers is a promising approach to optimize commercial phenotypes. Our results indicate that the intestinal microbiota profile could be reconstructed by external factors, leading to advantageous changes in muscle characteristics. The cecum microbiota of native broilers have the ability to improve certain meat quality and production performance. The population of *Lachnoclostridium* spp. could be used to regulate body weight and drip-losing rate in broilers, but more study is needed.

## Graphical Abstract

Experiment model for fecal Microbiota transplantation.
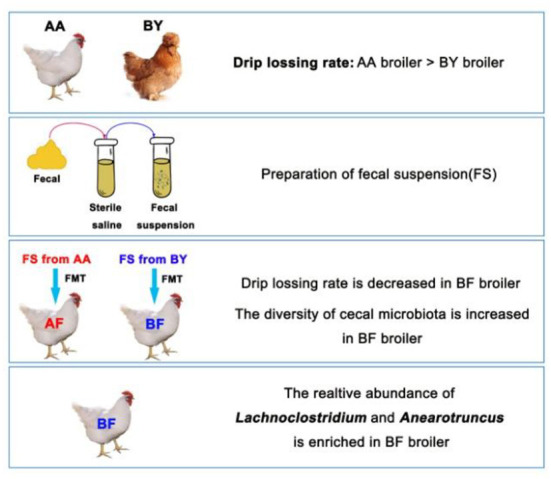

## Introduction

Fast-growing livestock has become a reliable protein source in the human diet. However, rapid growth induces lower proteolytic potential in muscle, which decreases the water-holding capacity of meat and ultimately leads to poor meat quality (1). Drip loss is regarded as a crucial factor to evaluate muscle water-holding capacity (2). Native broilers exhibit a lower drip loss in muscle than commercial broilers (3). Growing evidence shows that the muscle characteristics, fatty acid composition, and abdominal fat rate of native breed broilers were different from commercial breeds (4–6). These reports demonstrated that breed is an important factor in meat quality. Additionally, numerous studies have confirmed the existence of a muscle-gut microbiota axis (7, 8), suggesting a relationship between gut microbiota and muscle synthesis and metabolism. Therefore, regulating the gut microbiota of livestock has great potential to impact meat quality.

The intestinal microbiota of the broiler gastrointestinal tract are composed of billions of microbes that influence health and productivity (9). A recent study suggested that the regulation of the intestine *Clostridium* increased the composition of n-3 polyunsaturated fatty acids in the muscle of broilers (10). Other studies showed that the application of multispecies probiotics decreased drip loss in chicken muscle (11, 12). These reports demonstrate the existence of the intestinal microbiota–meat quality relationship, although the mechanism remains unclear. Many studies concerning meat quality have shown that muscle glycogen utilization, protein denaturation, degradation of muscle, and muscle fiber diameter and density affected the pH, water-holding capacity, and shearing force of meat (13–15). Additional studies concerning the gut microbiota-muscle axis showed that gut microbiota contributed to growth and the physiological state of muscle tissue (16), the substance and energy metabolism of muscle (17), as well as the synthesis and function of skeletal muscle (18). Therefore, the intestinal microbiota could be strategically regulated to improve meat quality through the muscle-gut microbiota axis.

In this study, we compared the gut microbiota composition between native broilers and commercial broilers. Our data reveal a remarkable distinction between the intestinal microbiota profiles of the Beijing-You broilers (a Chinese native chicken breed) and the Arbor Acres broilers (a commercial chicken breed). A mixed fecal suspension of the Arbor Acres broilers and the Beijing-You broilers was transferred to the Arbor Acres broilers through fecal microbiota transplantation (FMT). The reconstructed intestinal microbiota of the Arbor Acres broilers exhibited an upregulation in the expression of *MyoD1* gene of biceps femoris muscle, an increase in muscle fiber diameter, and a decrease in the drip loss. Thus, the meat quality was enhanced by the treatment. Bacteria 16S rDNA gene amplicon sequencing analysis was conducted and *Lachnoclostridium* spp. were identified as a potential microbes associated with meat quality and body weight in broilers. These findings indicate that meat quality can be improved through the regulation of intestinal microbiota.

## Materials and Methods

### Animals, Treatments, and Sampling

All the animals and breed eggs were supplied by the Beijing Broiler Breeding and Protection Base (Beijing Daxing district, China). All the animals were raised in the same environment. Fresh drinking water and a consistent diet were provided ad libitum. The diet was prepared according to the Nutrients Requirements of Chicken (NRC) (1994) without antibiotics and probiotics. Detailed information of the nutritional ingredients is shown in Supplementary Table S1.

### Microbiota Composition in Different Breeds of Broilers

Five fecal samples from the adult Arbor Acres broilers and five fecal samples from the adult Beijing-You broilers were selected randomly for 16s rDNA sequencing. The treatments were the Arbor Acres (**AA**) group and the Beijing-You (**BY**) group. Fifteen healthy Arbor Acres broilers and 15 Beijing-You broilers were selected as donors in next FMT experiment.

One-hundred 1-day-old male Arbor Acres chicks and 100 1-day-old male Beijing-You chicks were raised in a same environment until to 42 days of age. Six ileum fecal samples and six cecum fecal samples from the Arbor Acres chicks and the Beijing-You chicks were collected randomly for 16s rDNA sequencing weekly. At the age of 82 days, six intestinal content samples were collected from the Beijing-You broilers for 16s rDNA sequencing. When the age satisfied the commercial standard (42 days for the Arbor Acres broilers and 82 days for the Beijing-You broilers), six broilers in the two breeds broilers were selected to slaughter and the abdominal fat rate and drip loss of breast muscle and biceps femoris muscle were evaluated. The treatments were the Arbor Acres (**AA**) group and the Beijing-You (**BY**) group.

### Fecal Microbiota Transplantation

One hundred breeding eggs of the Arbor Acres broilers were incubated until born. All the eggshells were sterilized in advance with medicinal alcohol. Forty-four male 1-day-old newborn chicks were randomly assigned to two sterilized isolators and raised to 35 days of age. The chicks were incubated and raised in a sterile room with an air filtration system. Drinking water was sterilized by autoclave and feed was sealed and sterilized by electron beam radiation. Body weight was recorded every week. In first 21 days of the experiment, the following tasks were conducted everyday. Fresh feces from the two breed donors were collected to make a fecal suspension. Every chicken was provided 1 ml of the fecal suspension through oral gavage. At 35 days, five ileum contents and five cecum contents from each treatment group were selected randomly and stored in liquid nitrogen immediately. All of the intestinal content samples were stored at −80°C for further 16s rDNA sequencing. Abdominal fat was weighed. Breast muscle and biceps femoris muscle were collected and stored in a 4°C refrigerator for further analysis of meat quality. The treatments were as follows: the Arbor Acres fecal transferred into the Arbor Acres (**AF**) and the Beijing-You fecal transferred into the Arbor Acres (**BF**).

### Fecal Suspension From Donors

Twenty-five grams per treatment of spontaneously excreted feces were collected from the abovementioned donors (15 adult Arbor Acres broilers and 15 adult Beijing-You broilers). A total of 25 ml of stool suspension per treatment were prepared through filtration, centrifugation, and purification (19–21). A sterile syringe connected to the sterile infusion hose was used to conduct FMT for each chick inside the isolators. All the birds were colonized with 1 ml of fecal suspension everyday in the first 21 days of the experiment. From 1 to 3 days, florfenicol was added to drinking water.

### Meat Quality and Muscle Fiber Diameter

The formula to calculate abdominal fat rate is as follows: abdominal fat weight/body weight × 100%. To evaluate drip loss, muscle was cut into strips (1 cm × 1 cm × 5 cm) and weighed (W1) immediately after slaughter. Every muscle strip was wrapped in an airless bag and hung in a 4°C refrigeration house for 24 h. The entire muscle strip was suspended in the airless bag and did not touch the sides of the bag. After 24 h, the muscle strip weight was recorded as W2. The drip loss was calculated as follows: W2/W1 × 100% (22). Paraffin muscle sections were generated through Masson's trichrome staining and the muscle fiber diameter was measured by Image-Pro Plus software.

### Muscle Fiber Diameter-Related Gene Expression by Real-Time PCR

Briefly, muscle samples were frozen in liquid nitrogen and scored at −80°C. Total RNA extraction was conducted with TRIzol solution. RNA concentration was measured with a NanoDrop instrument (Thermo Fisher Scientific, USA) at 260 and 280 nm. Next, the total RNA was reverse-transcribed into complementary DNA (cDNA) by the StarScript II First-strand cDNA Synthesis Mix with guide DNA (gDNA) Remover (GenStar, Shanghai, China) and gene expression was determined by 2X RealStar Green Fast Mixture (GenStar, Shanghai, China) according to the protocol. The muscle fiber-related genes and β-actin primers are shown in Table 1, and 2–ΔΔCt expression was calculated to determine the related gene expression level (23). The primer sequence is shown in Table 1.

**Table 1:** Primer for reverse transcription-PCR (RT-PCR) amplification.

**Gene**			**Gene note**
MyoD1	Forward primer	CGACGGCATGATGGAGTACA	NM_204214.2
	Reverse primer	ATGCTTGAGAGGCAGTCGAG	
Myf5	Forward primer	CCTGAGGAAGAGGAACACGTC	NM_001030363.1
	Reverse primer	TTGGGGAGTCTCTGGTTGGG	
Myf6	Forward primer	GCACGGACTCTTTCCTACCC	NM_001030746.1
	Reverse primer	TGACAGGAATGGACTGCTCG	
MyoG	Forward primer	TGCAAGCGCAAAACCGTGTC	NM_204184.1
	Reverse primer	GCAGGATCTCCACCTTGGGC	
MSTN	Forward primer	GCCTGGAACAAGCACCTAAC	NM_001001461.1
	Reverse primer	AAGAGCCATCGCTACTGTCG	
β-actin	Forward primer	TCACCCAACACTGTGCCCATCTACGA	NM_001101.5
	Reverse primer	TCGGTGAGGATCTTCATGAGGTA	

### Total DNA Extraction, 16s RDNA MiSeq Sequencing, and Sequencing Data Analysis

#### Deoxyribonucleic Acid Extraction

Total DNA was extracted using the Omega E.Z.N.A. Stool DNA Kit (Omega Biotek, Incorporation, USA) following the manufacturer's instructions. The purity and quality of the genomic DNA were evaluated on 0.8% agarose gels.

#### Polymerase Chain Reaction Amplification

The V3-V4 hypervariable region of the bacterial 16S rRNA gene was amplified with the primers 338F (ACTCCTACGGGAGGCAGCAG) and 806R (GGACTACHVGGGTWCTAAT) (24). For each intestinal content sample, a 10-digit barcode sequence was added to the 5' end of the forward and reverse primers (Allwegene Company, Beijing, China). The PCR was conducted on the Mastercycler Gradient (Eppendorf, Germany) using 25 μl reaction volumes, containing 12.5 μl KAPA2G Robust HotStart ReadyMix, 1 μl forward primer (5 μM), 1 μl reverse primer (5 μM), 5 μl DNA (total template quantity of 30 ng), and 5.5 μl H_2_O. Cycling parameters were 95°C for 5 min, followed by 28 cycles of 95°C for 45 s, 55°C for 50 s, and 72°C for 45 s, with a final extension at 72°C for 10 min. Three PCR products per sample were pooled to mitigate reaction-level PCR biases. The PCR products were purified using the QIAquick Gel Extraction Kit (QIAGEN, Germany), quantified using real-time PCR, and sequenced.

#### High-Throughput Sequencing

Deep sequencing was performed on the MiSeq platform at Allwegene Company (Beijing, China). After the run, image analysis, base calling, and error estimation were performed using Illumina Analysis Pipeline version 2.6.

#### Data Analysis

The raw data were screened and sequences were removed from consideration, if they were shorter than 200 bp, had a low-quality score (≤20), contained ambiguous bases, or did not exactly match the primer sequences and barcode tags. Qualified reads were separated using sample-specific barcode sequences and trimmed with Illumina Analysis Pipeline version 2.6. The Quantitative Insights Into Microbial Ecology (QIIME) process was not directly used for operational taxonomic unit (OTU) clustering and species notes. Instead, we used some targeted software to deal with some pivotal steps (25, 26). Specifically, the UCHIME function in Vsearch software (version 2.7.1) was used to compare the sequences of removed chimeras. The UPARSE algorithm in Vsearch (version 2.7.1) was used for OTU clustering, according to 97% similarity (27). The annotated species were compared with the Silva 138 database of Basic Local Alignment Search Tool (BLAST) software and the species classification information corresponding to each OTU was obtained. The QIIME (version 1.8.0) software was used to analyze the alpha diversity index. The sequences were clustered into operational taxonomic units (OTUs) at a similarity level of 97% (27) to generate rarefaction curves and to calculate the richness and diversity indices. The ribosomal database project (RDP) classifier tool was used to classify all the sequences into the different taxonomic groups (28). The raw data were submitted in the sequence read archive (PRJNA771343 and PRJNA771493) of the National Center for Biotechnology Information (NCBI) for open access.

To examine the similarity between the different samples, clustering analyses and principal coordinate analysis (PCA) were performed, based on the OTU information from each sample and using R package (29). The evolutionary distances between microbial communities from each sample were calculated using the talc coefficient and represented as an unweighted pair group method with arithmetic mean (UPGMA) clustering tree describing the dissimilarity (1-similarity) between multiple samples (30). A Newick-formatted tree file was generated through this analysis. To compare the abundances and structure of microbial communities in the different samples, heat maps were generated with the top 20 OTUs using Mothur (31).

### Statistical Analysis

All the statistical analyses were performed using IBM SPSS version 19.0 software, except for the microbiota analysis. The one-way ANOVA was used to compare the differences among the experimental treatment group. The 16S rDNA sequencing data were analyzed by the Kruskal–Wallis and Tukey's tests to determine the significant differences. The difference was declared significant at *P* < 0.05 and a trend at 0.05 < *P* < 0.1 in all the analyses. Alpha diversity and beta diversity were tested using the data processing platform Allwegene Company (Beijing, China). The plots were visualized using R software packages.

## Results

### Different Breeds of Chicken Have Different Intestinal Microbiota Compositions

**Figure 1:**
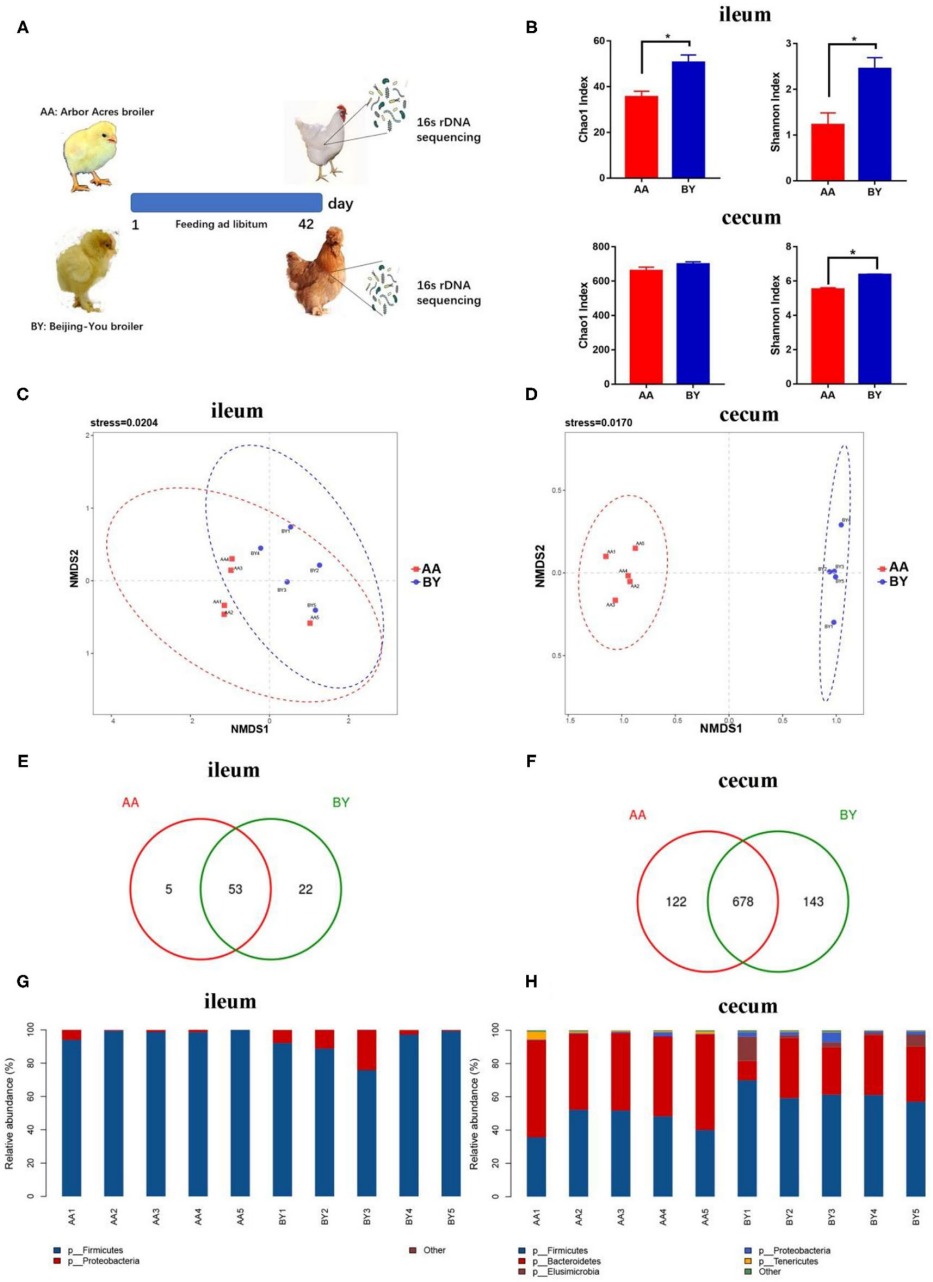
Intestinal microbiota structure of the Arbor Acres broilers and the Beijing-You broilers. **(A)** Graph of microbiota composition. **(B)** Alpha diversity analysis of the Chao1 index and the Shannon index. **(C,D)** Scatterplot from nonmetric multidimensional scaling (NMDS) in bacterial composition. **(E,F)** Venn diagram of unique operational taxonomic units (OTUs) in ileum and cecum. **(G,H)** Microbiota structure at the phylum level in ileum and cecum. AA: the Arbor Acres broilers; BY: the Beijing-You broilers. The numbers of independent biological samples analyzed in panel B to H were AA (*n* = 5) and BY (*n* = 5). Data in **(B)** are expressed as mean ± SEM. Data in **(B)** were analyzed using ANOVA followed by the Tukey's test and were considered as statistically significant at ^*^p < 0.05 between the indicated groups. Data in **(C,D)** were analyzed using nonmetric multidimensional scaling. Data in **(C–H)** statistical tests were two-sided and differences were considered to be statistically significant at *p* < 0.05.

Compared to the Arbor Acres broilers, the Beijing-You broilers had the higher Chao1 index and Shannon index, except for the Chao 1 index of cecum (Figures 1A,B). Nonmetric multidimensional scaling (NMDS) analysis showed that the remarkable difference in microbiota composition was species related (Figures 1C,D). More unique OTUs in ileum and cecum of the Beijing-You broilers were observed (Figures 1E,F). *Firmicutes* spp. was the most abundant bacteria in the ileum (Figure 1G). In the cecum of the Beijing-You (BY) broiler, more *Firmicutes* spp. and fewer *Bacteroidetes* spp. were found (Figure 1H). These results indicate unique intestinal microbiota compositions between the Arbor Acres broilers and the Beijing-You broilers, especially in the cecum.

### Changes in Microbiota Profiles of the Arbor Acres Broilers and the Beijing-You Broilers

**Figure 2:**
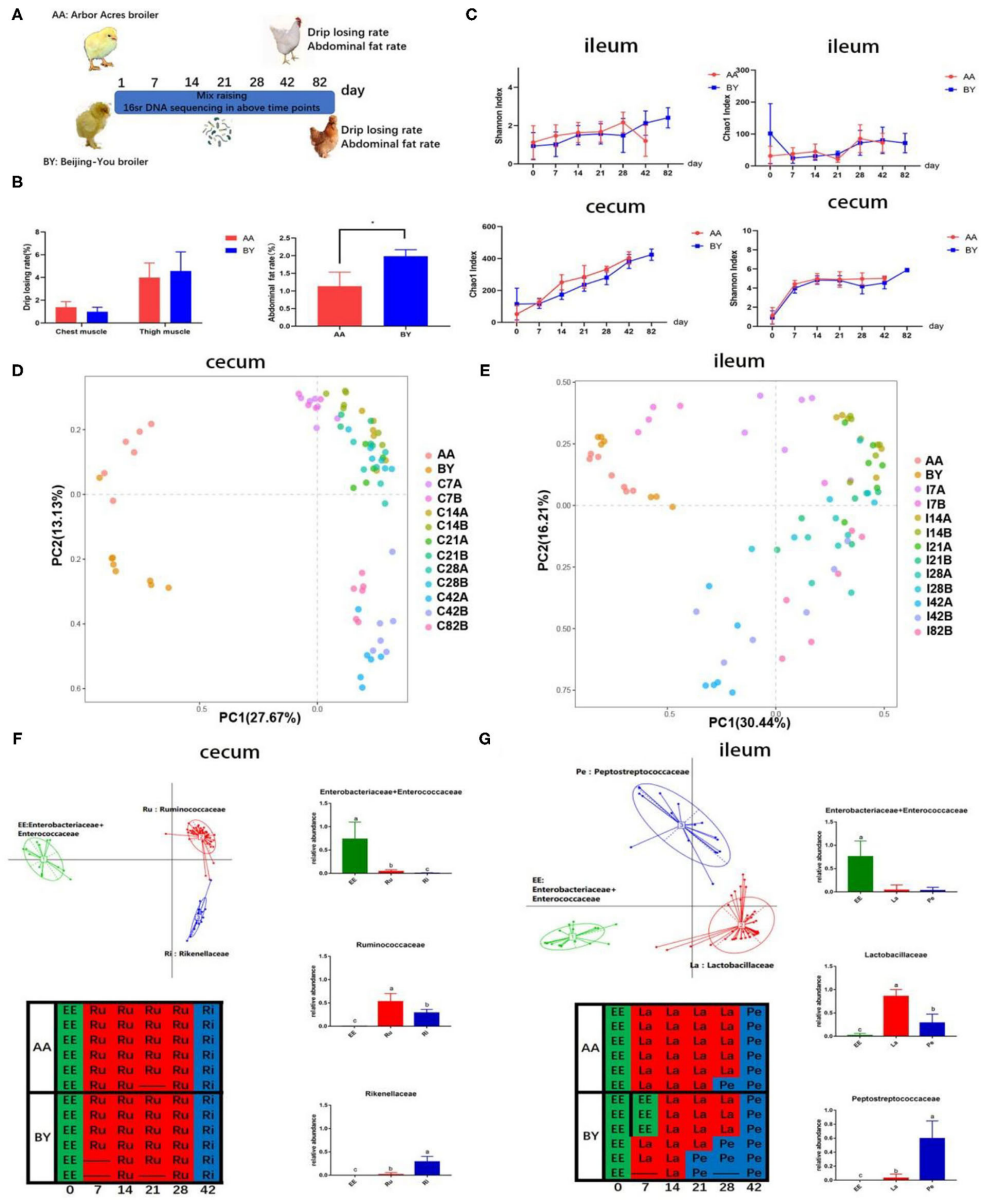
The changes in the microbiota of the co-raised Arbor Acres broilers and the Beijing-You broilers. **(A)** Graph of microbiota development. **(B)** Abdominal fat deposition and water-holding capacity of meat in the AA broilers and the BY broilers. **(C)** Alpha diversity analysis of the Chao1 and the Shannon indexes in the Arbor Acres broilers and the Beijing-You broilers. Principal component analysis (PCA) of the changes in intestinal microbiota in **(D)** cecum and **(E)** Partitioning around medoid (PAM) of enterotypes in F and G. Graph: enterotypes and dominant bacteria; Table: the temporal distribution of intestinal microbiota types. The numbers of independent biological samples analyzed in panel B to H were AA (*n* = 6) and BY (*n* = 6) and the numbers in panels of **(C–G)** were AA (*n* = 6) at each point and BY (*n* = 6) at each point. Data in **(B,C)** are expressed as mean ± SEM. Data in **(B,C)** were analyzed using ANOVA followed by the Tukey's test and were considered as statistically significant at *p < 0.05 between the indicated groups. Data in **(D,E)** were analyzed using PCA. Data in **(F,G)** were analyzed using PAM. Data in **(D–G)** statistical tests were two-sided and the differences were considered to be statistically significant at *p* < 0.05.

The alpha diversity of ileum and cecum was the same at each time point (Figure 2A). Remarkably, parallel of the developed rules of intestinal microbiota were discovered in the two breeds of broilers when the same feeding conditions were applied, according to the PCA results (Figures 2C,D). The same results were observed through principal coordinate analysis, which is based on the weighted and unweighted UniFrac distance (Supplementary Figure S3). To further understand the similarity in the development of the microbiota profiles, samples were collected at every time point and analyzed by a partitioning around method to distinguish intestinal microbiota types during the experiment (Figures 2E,F). Intestinal microbiota types of EE (*Enterobacteriaceae* and *Enterococcaceae*), Ru (*Ruminococcaceae*), and Ri (*Rikenllaceae*) were enriched in cecum (Figure 2E). The selected bacteria occupied the dominant position in respective intestinal microbiota types (Figure 2E). The distribution of intestinal microbiota types over time for the Arbor Acres (AA) broilers and the Beijing-You (BY) broilers was exactly the same (Figure 2E). Similarly, intestinal microbiota types of EE (*Enterobacteriaceae* and *Enterococcaceae*), La (*Lactobacillaceae*), and Pe (*Peptostreptococcaceae*) were observed in the ileum. The distribution regularity of ileum microbiota types was nearly same, except for individual samples, compared to the AA broilers and the BY broilers (Figure 2F). These results indicate that the microbiota profile was converged although even among different breeds of the broiler. The Beijing-You broilers had more abdominal fat, which is a marker of fat deposition in chicken (Figure 2B). However, no difference was observed in the drip loss of the muscle, which reflects the water-holding capacity of meat (Figure 2B). The same regular of microbiota components may induce drip loss, which is a breed-related phenotype, to become semblable.

### Fecal Microbiota Transplantation Induces a Shift in the Cecum Microbiota Composition of the Arbor Acres Broilers

**Figure 3:**
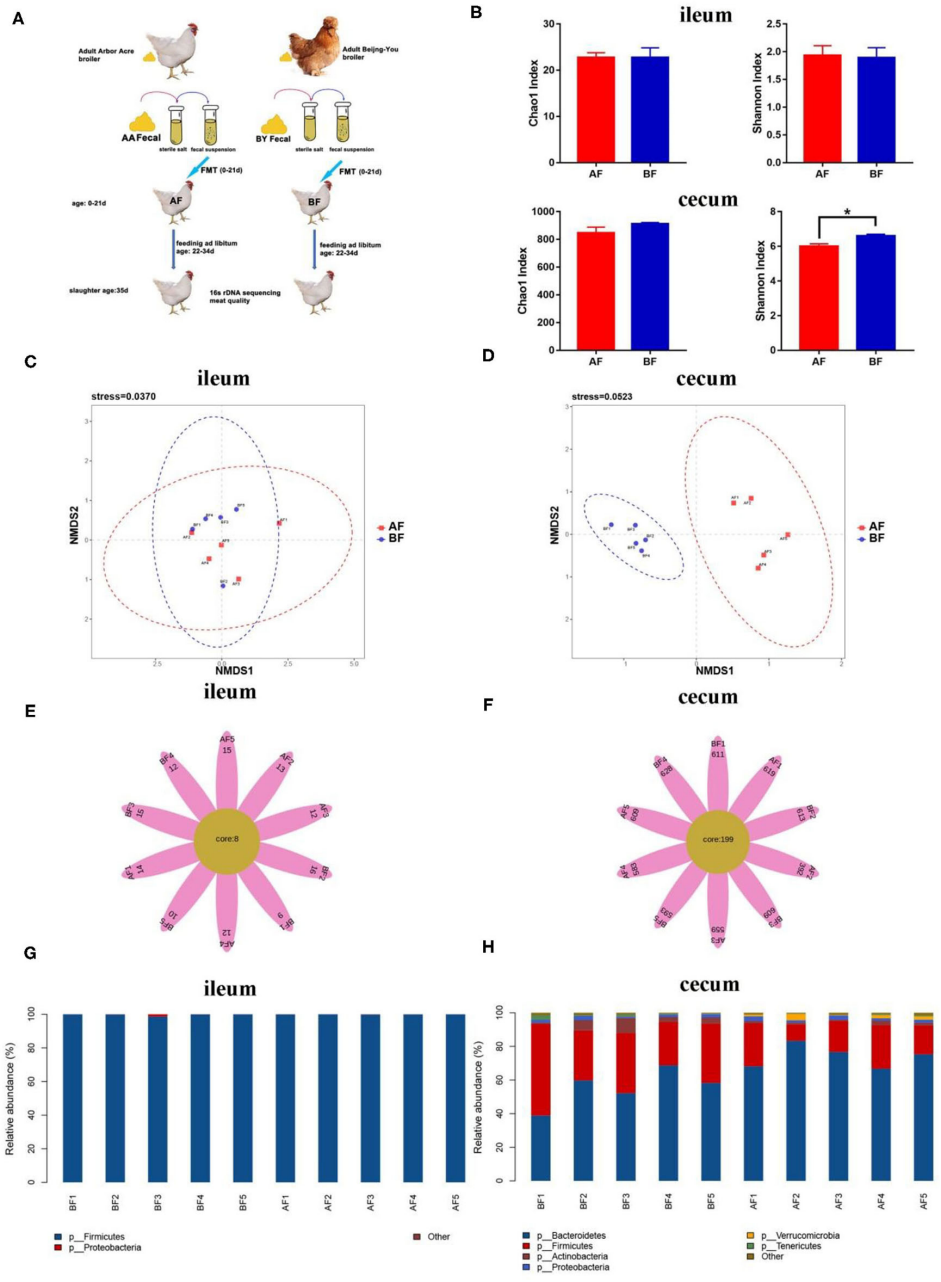
16s rDNA sequencing analysis of different breeds in ileum and cecum after fecal microbiota transplantation (FMT). **(A)** Graph of FMT experiment. **(B)** Alpha diversity analysis of the Chao1 index and the Shannon index. **(C,D)** Scatterplot from NMDS in bacterial composition. **(E,F)** Venn flower diagram of core OTUs in ileum and cecum. **(G,H)** Intestinal microbiota structure of ileum and cecum at the phylum level. AF: Fecal transfer from the Arbor Acres broilers into the Arbor Acres broiler; BF: fecal transfer from the Beijing-You broilers into the Arbor Acres broilers. The numbers of independent biological samples analyzed in panel B to H were AF (*n* = 5) and BF (*n* = 5). Data in **(B)** are expressed as mean ± SEM. Data in **(B)** were analyzed using ANOVA, followed by the Tukey's test, and were considered as statistically significant at *p < 0.05 between the indicated groups. Data in **(C,D)** were analyzed using nonmetric multidimensional scaling. Data in **(C–H)** statistical tests were two-sided and the differences were considered to be statistically significant at *p* < 0.05.

To confirm the relationship between intestinal microbial changes and drip loss, an FMT experiment was designed. Highly abundant bacterial species were identified in cecum of BF, as indicated by the Shannon index (Figures 3A,B). Few unique OTUs were found in ileum, but numerous unique OTUs were discovered in cecum (Figures 3E,F). NMDS analysis indicated two independent bacteria clusters in the cecum of the receptor group (Figures 3C,D). These results indicate prominently different microbiota species existed in the cecum of receptor animals through FMT. *Firmicutes* spp. was the primary species in ileum between the two groups (Figure 3G). More *Firmicutes* and fewer *Bacteroidetes* were found in the cecum of the BF group (Figure 3H). These results (Figure 3) are similar to that of the Arbor Acres (AA) broilers and the Beijing-You (BY) broilers raised in a common environment (Figure 1).

### Phenotypes Associated With Meat Quality and Production Performance Were Altered Through FMT

**Figure 4:**
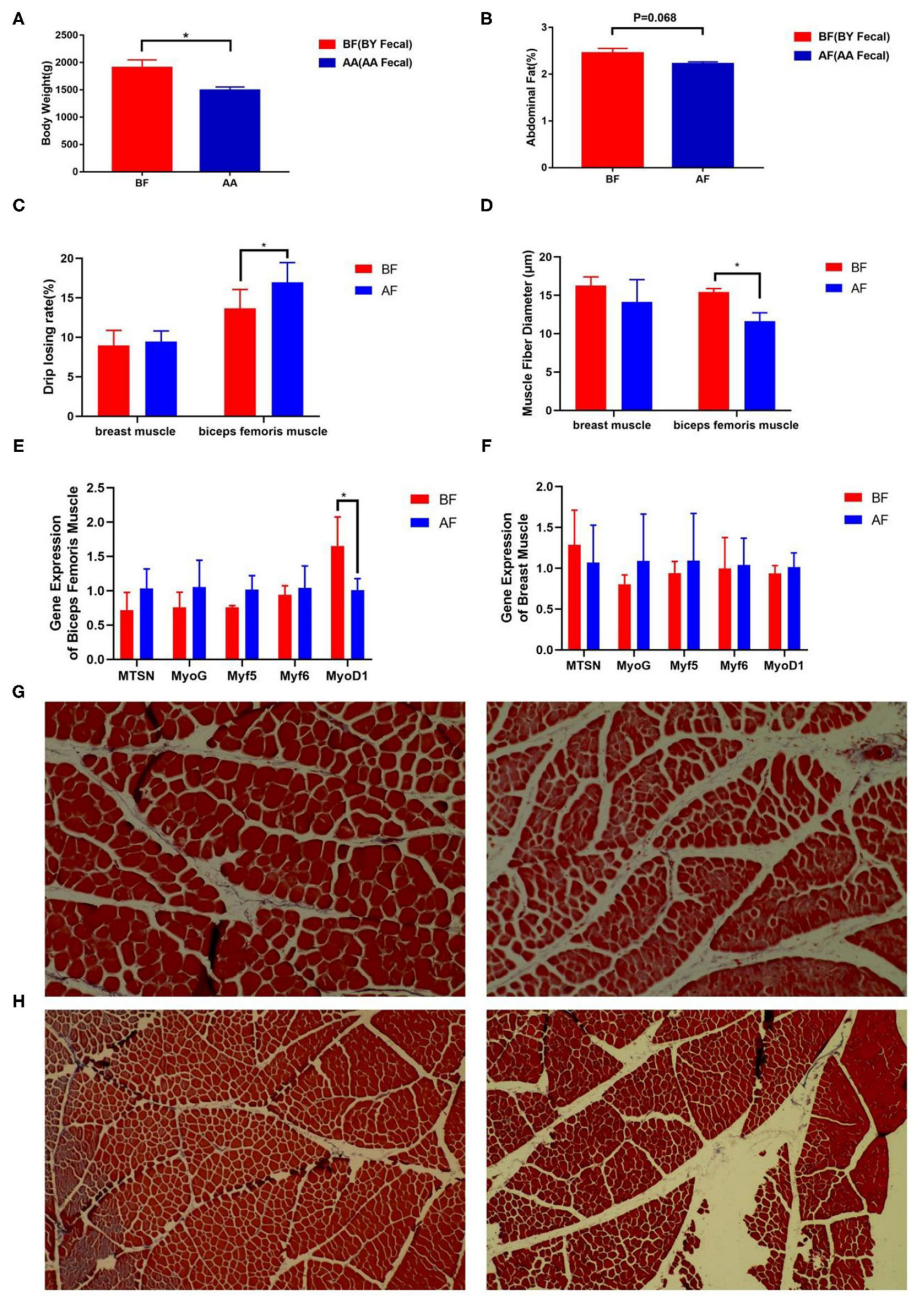
Drip loss and abdominal fat were altered after FMT. **(A)** Body weight, **(B)** abdominal fat, **(C)** drip loss, **(D)** muscle fiber diameter after FMT, **(E,F)** relative expression of muscle fiber diameter-related genes, **(G,H)** paraffin sections of muscle fiber (BF: left and AF: right) [**(G)** biceps femoris muscle fiber and **(H)** breast muscle fiber]. AF: Fecal transfer from the Arbor Acres broilers into the Arbor Acres broiler; BF: Fecal transfer from the Beijing-You broilers into the Arbor Acres broilers. The numbers of independent biological samples analyzed in panel A to H were AF (*n* = 5) and BF (*n* = 5). All the data are expressed as mean ± SEM. All the data were analyzed using ANOVA, followed by the Tukey's test, and were considered as statistically significant at *p < 0.05 between the indicated groups. Figures in **(G,H)** are muscle fiber of HandE staining.

To track the difference in phenotypes after FMT, body weight and meat quality index were measured. The body weight (*p* < 0.05) and abdominal fat rate (*p* = 0.068) in BF were higher than those in AF (Figures 4A,B). Lower drip loss (*p* = 0.067) and higher muscle fiber diameter (*p* < 0.05) of thigh muscle were found in BF (Figures 4C,D). Five genes that regulate muscle fiber diameter synthesis were selected, but only the relative expression of MyoD1 in BF biceps femoris muscle significantly higher than that in AF (Figure 4E). The difference in muscle fiber diameter was visually reflected through paraffin sections (Figure 4G). There was no difference in the meat quality of the chest muscle (Figures 4C,D,F,H). These results indicate intestinal microbiota decreased drip loss by increasing MyoD1 expression in broilers and this characteristic can be transferred from FMT donor to receptor.

### *Lachnoclostridium* Associated With Drip Loss and Body Weight Could Be Used to Regulate Meat Quality and Production Performance

**Figure 5:**
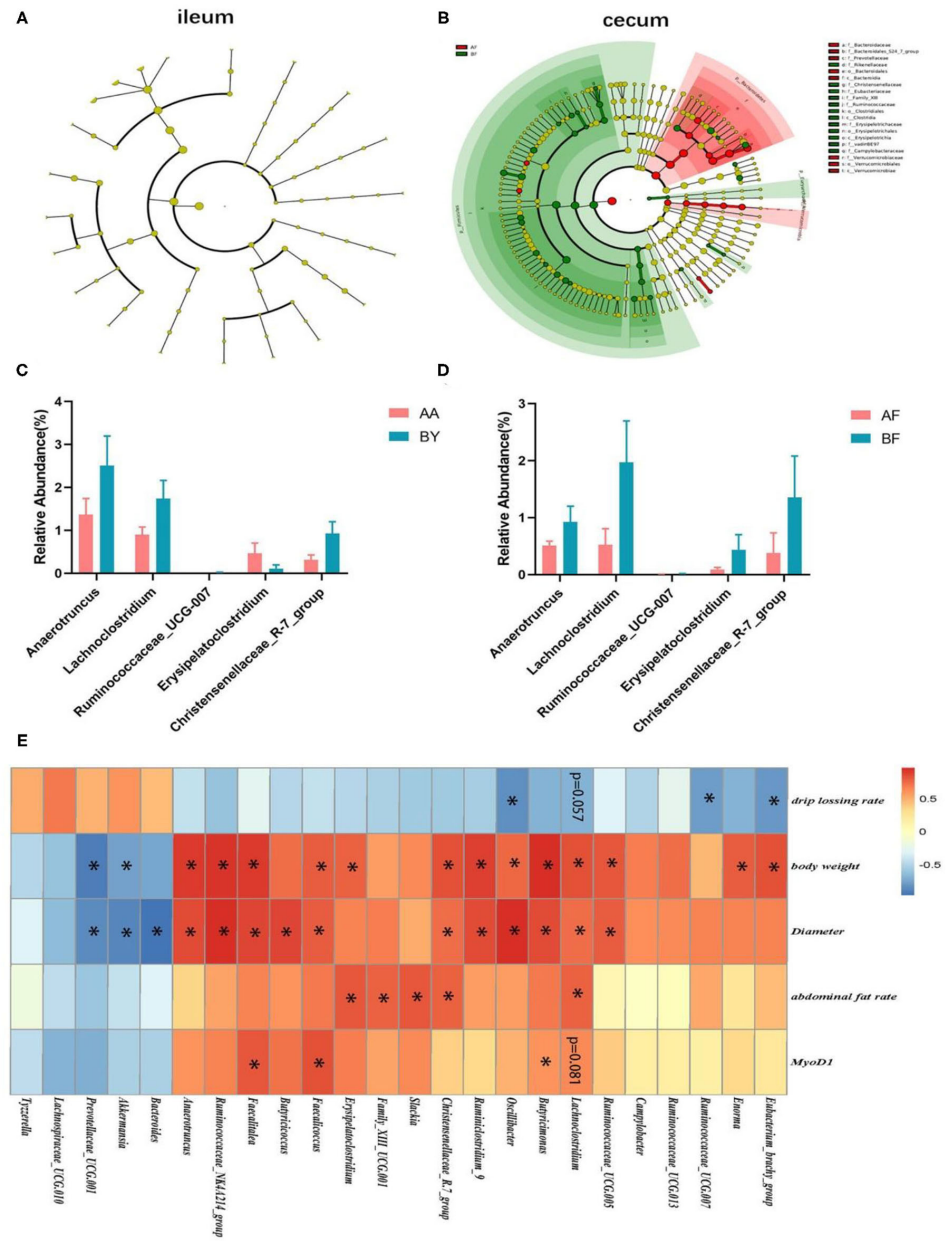
Analysis of the correlation between cecum intestinal bacteria at the genus level and phenotypes. **(A,B)** Cladograms of different microbiota in the ileum and cecum in recipients. **(C,D)** Bacteria (genus level) discovered in the cecum of both the donors **(C)** and receptors **(D)**; **(E)** The heat map of the correlation analysis between different bacteria in cecum and phenotypes. AF: Fecal transfer from the Arbor Acres broilers into the Arbor Acres broiler; BF: Fecal transfer from the Beijing-You broilers into the Arbor Acres broilers. The numbers of independent biological samples analyzed in **(A–H)** were AF (*n* = 5) and BF (*n* = 5). All the data are expressed as mean ± SEM. All the data were analyzed using ANOVA, followed by the Tukey's test, and were considered as statistically significant at *p < 0.05 between the indicated groups.

The Wilcoxon analysis of different bacteria in donors is shown in Supplementary Figure S3. Most of the different microbiota existed in the cecum, while some were found in the ileum (Supplementary Figure S2). Cladogram data show that different microbes including *Firmicutes* and *Bacteroidetes* were discovered in the cecum, but none were found in the ileum (Figures 5A,B). Donors and recipients exhibited the same trend in the ratio of relative abundance of *Firmicutes* and *Bacteroidetes* (Supplementary Figure S1). At the genus level, *Lachnoclostridium, Anaerotruncus, Ruminococcaceae-UCG-007*, and *Christensenellaceae_R-7_group* were found in both the donors and recipients, with similar trends in relative abundance (Figures 5C,D). Seventeen genera and 14 genera were associated with body weight and biceps femoris muscle fiber diameter, respectively. Five genera and three genera were related to abdominal fat and drip loss, respectively. Three genera were associated with the relative expression of MyoD1 (Figure 5E). The statistical results of the correlation analysis are shown in Supplementary Table S2. According to Supplementary Table S2, the abundance of *Lachnoclostridium* spp. was positively correlated with body weight, abdominal fat rate, and biceps femoris muscle fiber diameter. Additionally, the abundance of *Lachnoclostridium* spp. was negatively correlated with drip loss and positively correlated with the relative expression of MyoD1. Interestingly, *Lachnoclostridium* was the only genera that exhibited a significant correlation trend with all the phenotypes. *Anaerotruncus* was positively correlated with body weight and muscle fiber diameter. *Christensenellaceae_R-7_group* was positively correlated with body weight, abdominal fat rate, and muscle fiber diameter (Figure 5E). These results indicate that cecum microbiota are associated with production performance and meat quality. *Anaerotruncus, Christensenellaceae_R-7_group*, and, in particular, *Lachnoclostridium* may serve as indicators of improved meat quality and production performance.

## Discussion

The quality of meat is of particularly importance to consumers and agricultural industries (32). Native breeds of livestock have superior meat quality and capacity for fat deposition, compared to commercial breeds (33, 34). Recently, numerous studies have demonstrated the important role of gut microbiota in regulating skeletal muscle synthesis and development (26, 35, 36). The gut microbiota-muscle axis has been identified (37, 38) and, to the best of our knowledge, this is the first report on the intestinal microbiota profile of the Beijing-You broilers by FMT. Moreover, we aimed to clarify the relationship between microbiota regulation and muscle development, with the goal of improving meat quality. We first compared difference in the bacterial composition of gut microbiota profiles between the Beijing-You broilers and the Arbor Acres broilers. Our data show a significant distinction in the intestinal microbiota structure between native broilers and commercial broilers. A recent study suggests that in contrast to mammals, poultry genotypes play a small role in shaping intestinal bacterial structure (39). Other studies have shown that genotypes play a decisive role in only a small fraction of the microbiota composition (40). However, growing evidence has demonstrated that gut microbiota have a decisive role in the interaction of host physiology (41). We suspected the intestinal microbiota composition, the regulation of intestinal microbiota development, and some microbe-regulated phenotypes could be changed by external factors.

We tracked the development of intestinal microbiota in the Arbor Acres broilers and the Beijing-You broilers. In a same feeding environment, the Arbor Acres broilers and the Beijing-You broilers were raised together and over a period of 42 days, their microbiota became remarkably similar. In a subsequent FMT experiment, the gut microbiota compositions of the Arbor Acres broilers changed significantly via FMT treatment. These results suggest that external factors could be used to shape and influence the intestinal microbiota structure in broilers. In addition, recent reports have shown nutrition to be an important factor for rebuilding intestinal microbiota structure in broilers (42, 43). Another study indicated that the drip loss of native broilers is significantly different from commercial broilers (44). Interestingly, through same environmental conditions and FMT treatment, the muscle of the Arbor Acres broilers and the Beijing-You broilers exhibited a similar drip-loss rate. These results suggest the feasibility of regulating meat quality through strategic changes to the intestinal microbiota.

To elucidate the relationship and possible mechanism between gut microbiota and drip loss, we designed an FMT experiment. Obvious differences in the gut microbiota profile were observed between the BF broilers and the AF broilers. Oral administration of a fecal suspension from the Beijing-You broilers decreased the drip-loss rate of the Arbor Acres broilers, suggesting the gut microbiota derived from native broilers could be used to improve the meat quality of commercial breeds. Compared to commercial broilers, some studies have shown that native broilers have lower drip-loss rates and longer muscle fiber diameter (3, 44, 45). Consistent with these reports, our data show that oral fecal microbes decreased drip loss and an increased biceps femoris muscle fiber diameter of the Arbor Acres broilers, suggesting that certain muscle characteristics may be influenced by the gut microbiota from native broilers to commercial broilers. We assumed that the increase in muscle fiber diameter may be caused by the acceleration of muscle synthesis; therefore, we focused on the gene expression involved in muscle cell development. We discovered higher gene expression of MyoD1 in the Arbor Acres broilers after oral administration of the fecal suspension from the Beijing-You broilers. *MyoD* genes are considered as candidate genes for meat production traits in livestock (46). MyoD1 promotes the differentiation of muscle precursor cells and the proliferation of muscle cells (47), increasing the muscle fiber diameter (48, 49). Thick myofilament and thin myofilament of myofibril cross to form a lattice structure that provides space for water storage. The water stored in the filament lattice is lost after butchering because of muscle shrinking (50, 51). Moreover, muscle fiber diameter has been shown to decrease during tetanic development after butchering (52) because the shrinkage of myofibrils leads to a shorter lateral distance of cells and compresses water holding the space (53). Conversely, higher muscle fiber diameter may increase the capillary tubular structure, which increases the water-holding capacity and, in turn, decreases the drip loss. Muscles contain about 75% water, which is easily lost during slaughter, processing, and storage (51). Thus, more water storage space supported by longer muscle fiber diameter ensures greater water-holding capacity. In this study, FMT treatment increased the gene expression of MyoD1 to increase biceps femoris muscle fiber diameter and ultimately reinforced the water-holding capacity of muscle. Some specific genera of bacteria in the Beijing-You broilers may play a key role in regulating muscle characteristic.

Next, we aimed to identify key bacterial genera by correlation analysis. *Ruminococcaeae* and *Lachnospiraceae* were negatively correlated to drip loss and these genera play key roles in producing butyric acid and acetic acid (54, 55). At the genus level, the microbiota in the BF broilers exhibited an increased abundance in *Lachnoclostridium* and *Anaerotruncus* and this result was almost the same as the BY broilers. Species of *Lachnoclostridium*, belonging to Lachnospiraceae, has the ability to breakdown a wide variety of indigestible polysaccharides and ferment dietary fiber for the host via the production of butyric acid and acetic acid (56, 57). *Anaerotruncus* was discovered in human feces and revealed to produce acetic acid and butyric acid (58). In addition, *Lachnoclostridium* in cecum was the only genus associated with all the phenotypes in this study. Thus, *Lachnoclostridium* has the potential to regulate and be an indicator of meat quality in broilers. *Lachnoclostridium* and *Anaerotruncus* may enhance the absorption of volatile fatty acids to increase the energy utilization of recipients, leading to an increased muscle fiber diameter and decreased drip loss.

In this study, the body weight and abdominal fat rate of the BF broilers increased compared to the AF broilers. Higher relative abundance of *Firmicutes* and lower relative abundance of *Bacteroidetes* were both discovered in the BF broilers. Some studies have shown that more *Firmicutes* and fewer *Bacteroidetes* indicate higher energy efficiency for the host (59). High energy efficiency contributes to fat deposition of the host. Our results indicate that the microbiota composition of the BF broilers may increase body weight and abdominal fat deposition by enhancing energy utilization efficiency of the host. We also found that a high abundance of *Lachnoclostridium* was associated with high body weight. Some studies have shown that when body weight increased, the relative abundance of *Lachnoclostridium* in the cecum increased (60). In addition, our results indicate that all the bacteria at the genus level that were positively correlated with muscle fiber diameter were derived from *Firmicutes* and most of the genera that were negatively correlated with muscle fiber diameter were belonged to *Bacteroidetes*. Some reports have indicated a significant interaction between energy level and muscle fiber diameter (61) and high energy dietary contributed to an increasing muscle fiber diameter (62). High energy utilization efficiency may be contributed to an increased muscle fiber diameter. *Lachnoclostridium* may play an important role in energy efficiency and muscle fiber diameter; however, more studies are required to elucidate the specific mechanism in further experiments.

Some limitations of this study should be noted. The sample numbers of each group were limited, although all the samples were selected randomly in the hatchery. We acknowledge that a better result may exist with a larger sample size. Simple qPCR results may limit on experimental accuracy, while there had been ample reports of our results. Culturing bacteria *in vitro* and validation experiments *in vivo* are needed. Further study on the mechanism between microbiota and meat quality is also necessary.

## Conclusion

This study revealed a significant distinction between the intestinal microbiota composition of the Beijing-You broilers and the Arbor Acres broilers. This distinction is more greatly affected by external factors than genetics. The alteration of intestinal microbiota induced certain changes in drip loss by regulating muscle fiber diameter. Some specific cecum microbiota of native broilers may serve as indicators of meat quality, but more study is necessary. These findings provide a new strategy to optimize meat quality and theoretical evidence for the existence of the microbiota-muscle axis.

## Data Availability Statement

The datasets presented in this study can be found in online repositories. The names of the repository/repositories and accession number(s) can be found at: https://www.ncbi.nlm.nih.gov/sra; PRJNA771343 and PRJNA771493.

## Ethics Statement

The animal study was reviewed and approved by China Agricultural University Animal Care and Use Committee (AW81601202-1-1).

## Author Contributions

BZ contributed to the assay design and the optimization of the experimental design and conceived the project, obtained funding, and supervised the study. JLe and JLi sourced and/or collected specimens for testing, assisted with the experimental design, and prepared manuscript drafts. QH contributed to assay design, performed experiments, analyzed data, prepared libraries for sequencing, and edited manuscript drafts. YH contributed to samples collection and assisted with experimental design. YL contributed to the assay design. YD and YK contributed to the project conception, project supervision, and manuscript revision. All authors contributed to the article and approved the submitted version.

## Funding

This study was supported by the National Key R&D Program of China (2018YFE0127300) and the 2115 Talent Development Program of China Agricultural University.

## Conflict of Interest

YK was the employed of Kawamura & Co., Ltd. The remaining authors declare that the research was conducted in the absence of any commercial or financial relationships that could be construed as a potential conflict of interest.

## Publisher's Note

All claims expressed in this article are solely those of the authors and do not necessarily represent those of their affiliated organizations, or those of the publisher, the editors and the reviewers. Any product that may be evaluated in this article, or claim that may be made by its manufacturer, is not guaranteed or endorsed by the publisher.
