# Supplementary material for: Intestinal Microbiota Regulate Certain Meat Quality Parameters in Chicken
Source: Front Nutr. 2022 Apr 25;9:747705. doi: 10.3389/fnut.2022.747705 (PMC9085416; doi:10.3389/fnut.2022.747705)
Supplement: Supplementary file 1 [file Data_Sheet_6.pdf]

SUPPLEMENTARY DATA

Table S1. Diet composition and nutrition levels

Ingredients	d1-21%	d22-35%
Corn	55.08	61.071
Soybean meal	38.321	32.716
Plant oil	3.113	2.886
Calcium bicarbonate	1.265	1
Stone powder	1.362	1.35
NaCl	0.25	0.25
Microelement premix*	0.2	0.2
Vitamin premix**	0.03	0.03
DL-methionine	0.184	0.188
L-lysine hydrochloride	0.06	0.015
Choline chloride	0.1	0.174
Phytase(5000D)	0.015	0.1
Santoquin	0.02	0.02
Total	100	100
Nutrient Level***		
MC kj/kg	2.95	2.99
CP g/kg	21.4	19.5
Lys g/kg	1.2	1.15
Met g/kg	0.49	0.47
Ca g/kg	0.95	0.9
Available phosphors g/kg	0.45	0.4

*Microelement remix provides following per kg diet: Cu, 8mg ($CuSO_4 \cdot 5H_2O$); Fe, 80mg ($FeSO_4$); Mn, 100mg ($MnSO_4 \cdot H_2O$); Se, 0.15mg (Na_2SeO_3); I, 0.35mg (KI). **Vitamin premix provides following per kg diet: VA, 9500IU; VD3, 62.5ug; VE, 30IU; VK3, 2.65mg; VB1, 2mg; VB6, 2mg; VB12, 0.025mg; Biotin, 0.0325mg; Folic Acid, 1.25mg; Pantothenic Acid, 12mg; Nicotinic Acid, 50mg. ***Nutrient Level：Calculated accoroding to NRC(1994).

Table S2 Statistical result of correlation analysis between difference microbiota and phenotypes (p-value)

	body weight	abdominal fat rate	drip losing	MyoD1	Diameter
Butyricicoccus	0.055	0.148	0.223	0.040	0.004
Oscillibacter	0.043	0.182	0.008	0.202	0.000
Tyzzerella	0.273	0.719	0.182	0.249	0.433
Prevotellaceae_UCG-001	0.004	0.166	0.216	0.055	0.006
Faecalicoccus	0.027	0.075	0.291	0.007	0.015
Erysipelatoclostridium	0.032	0.024	0.207	0.055	0.070
Family_XIII_UCG-001	0.177	0.035	0.177	0.145	0.060
Lachnospiraceae_UCG-010	0.143	0.298	0.051	0.059	0.081
Enorma	0.027	0.575	0.056	0.418	0.070
Butyricimonas	0.001	0.074	0.064	0.105	0.006
Ruminococcaceae_UCG-005	0.023	0.842	0.432	0.337	0.016
Akkermansia	0.048	0.343	0.100	0.171	0.004
Lachnoclostridium	0.017	0.037	0.057	0.081	0.034
Anaerotruncus	0.003	0.426	0.277	0.108	0.004
Ruminococcaceae_UCG-007	0.280	0.220	0.020	0.701	0.063
Ruminococcaceae_NK4A214_group	0.001	0.189	0.109	0.067	0.000
Christensenellaceae_R-7_group	0.018	0.034	0.143	0.351	0.029
Campylobacter	0.088	0.943	0.193	0.668	0.092
Faecalitalea	0.002	0.100	0.501	0.011	0.004
Slackia	0.106	0.020	0.134	0.083	0.181
Ruminiclostridium_9	0.006	0.178	0.159	0.419	0.004
Bacteroides	0.055	0.483	0.243	0.178	0.000
Ruminococcaceae_UCG-013	0.054	0.955	0.539	0.643	0.074
Eubacterium_brachy_group	0.015	0.292	0.015	0.566	0.069
Ruminococcus_torques_group	0.002	0.621	0.342	0.228	0.019

Table S3. Correlation analysis of drip losing rate and cecum microbiota (top 20 except for uncluster, unidentify, others)

	breast muscle		biceps femoris muscle	
	Correlation value	P-value	Correlation value	P-value
Alistipes	-0.170401972	0.59647	0.563682067	0.056296
Faecalibacterium	-0.009634092	0.97629	-0.152937204	0.635124
Ruminococcus_torques_group	-0.15195048	0.63733	-0.338450081	0.281891
Bacteroides	-0.033505494	0.91767	0.036045746	0.911447
Barnesiella	0.227777152	0.47646	-0.035532052	0.912705
Ruminococcaceae_group_004	0.009485796	0.97666	-0.078942356	0.807333
Anaerotruncus	-0.167984197	0.60177	-0.009496264	0.976633
Lactobacillus	0.009864106	0.97573	-0.227056147	0.4779
Lachnoclostridium	-0.321680701	0.30791	-0.061676258	0.848986
Eisenbergiella	-0.485988575	0.10918	0.136798658	0.671607
Subdoligranulum	-0.045108795	0.88929	-0.11867173	0.713371
Streptococcus	0.031389765	0.92285	0.231876464	0.468345
Butyricicoccus	0.196370595	0.54074	-0.404842798	0.191738
Ruminococcaceae_group_005	0.024293425	0.94026	-0.112127693	0.728634
Flavonifractor	-0.338661115	0.28157	0.268252829	0.399222
Coprococcus1	0.221934751	0.48815	-0.277575591	0.382389
Oscillibacter	-0.099132145	0.75921	-0.357251049	0.254271

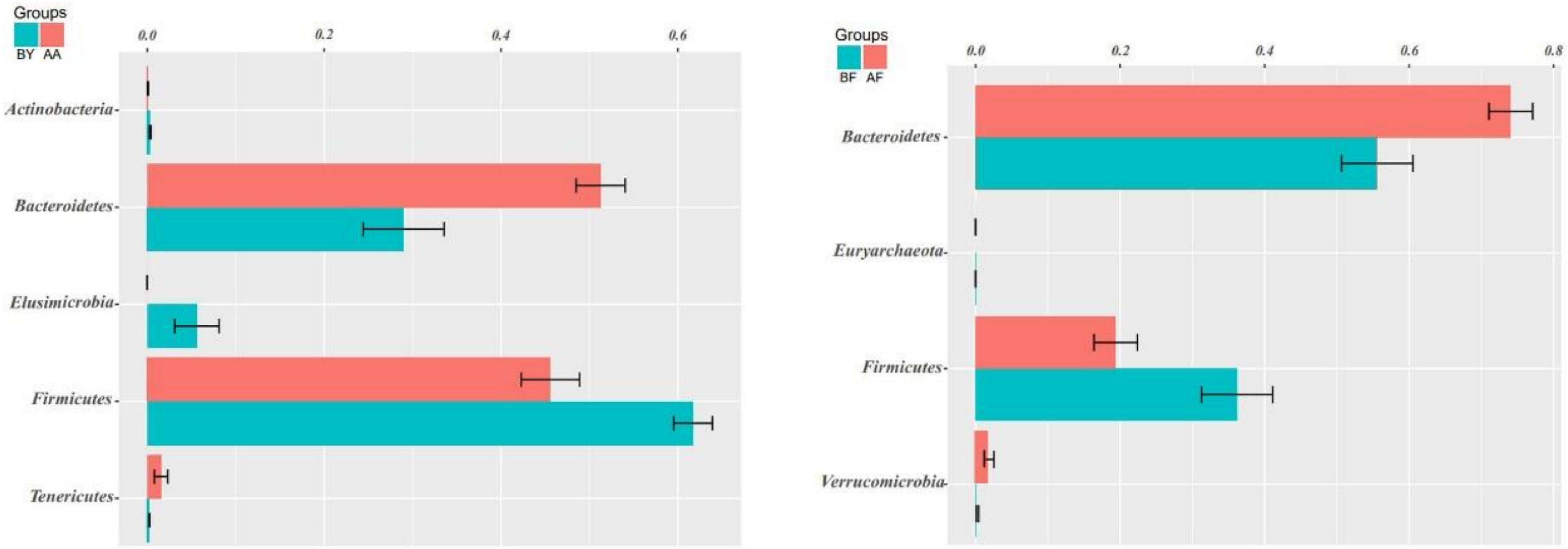

Figure S1The relative abundance of different cecum intestinal bacteria (phylum level) in donators and recipients

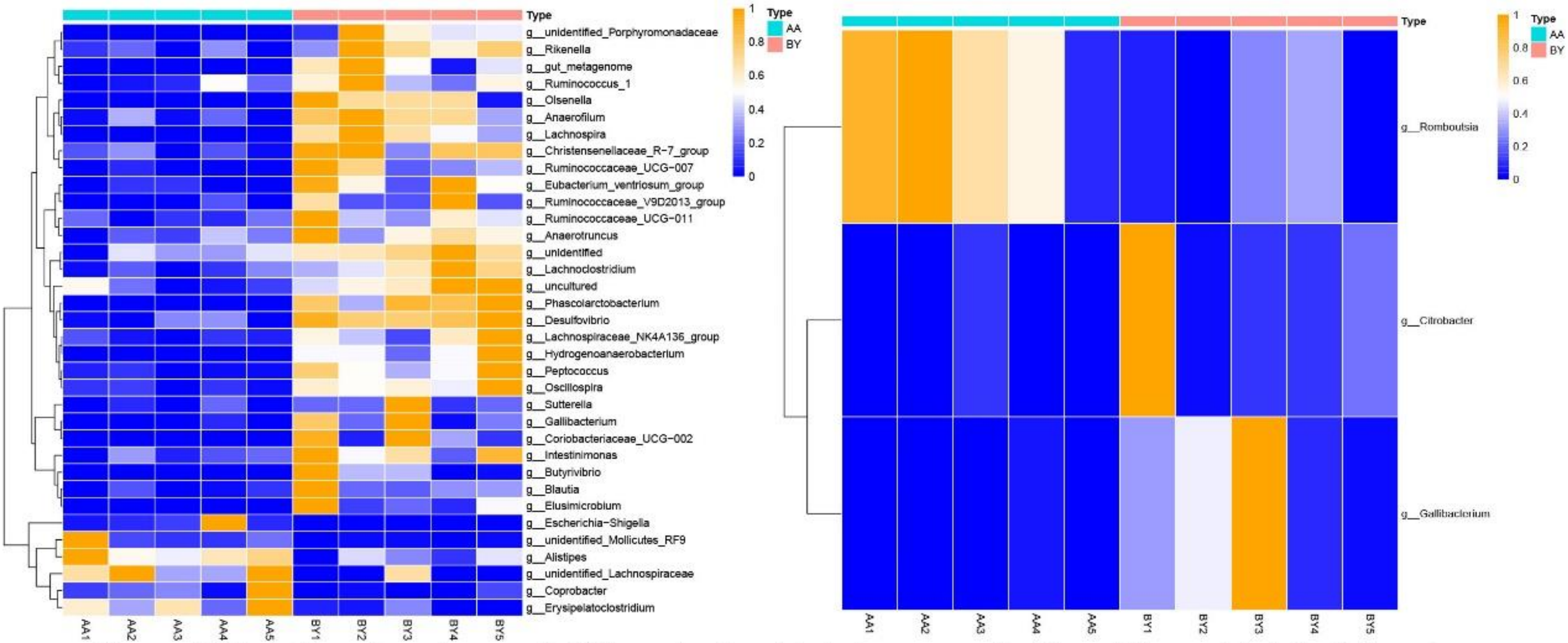

Figure S2 Relative abundance of different microbiota in cecum (left) and ileum (right) of donators

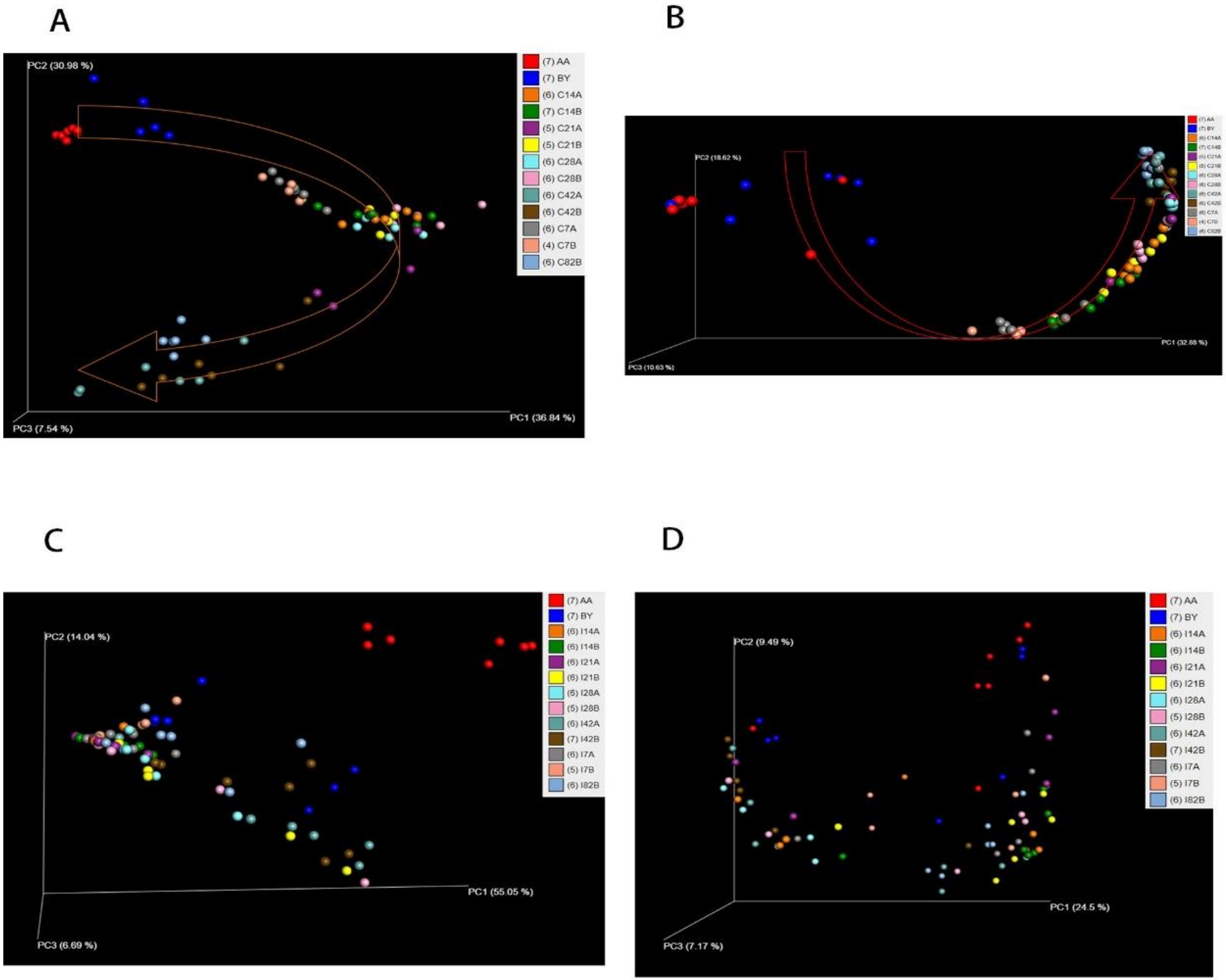

Figure S3 Develop regularity of intestinal microbiota according to analysis of PCOA. (A) PCOA of weighted unifrac distance in cecum. (B) PCOA of unweighted unifrac distance in cecum. (C) PCOA of weighted unifrac distance in ileum. (D) PCOA of unweighted unifrac distance in ileum.
